# Supplementary figures and images for: A comprehensive analysis of transcriptomic data for comparison of plants with different photosynthetic pathways in response to drought stress
Source: PLoS One. 2023 Jun 27;18(6):e0287761. doi: 10.1371/journal.pone.0287761 (PMC10298789; doi:10.1371/journal.pone.0287761)

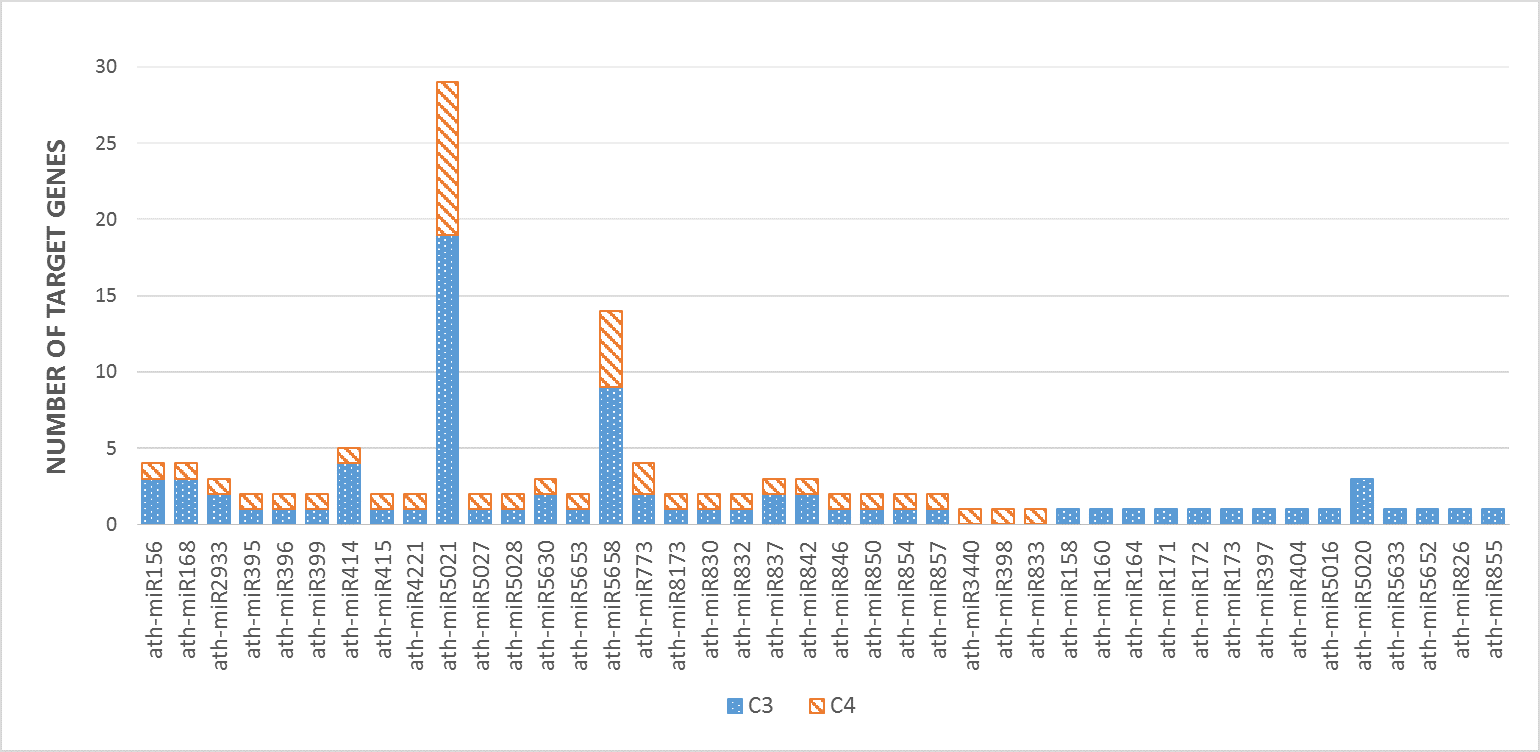

Supplement: S1 Fig — (TIF) [file pone.0287761.s001.tif]

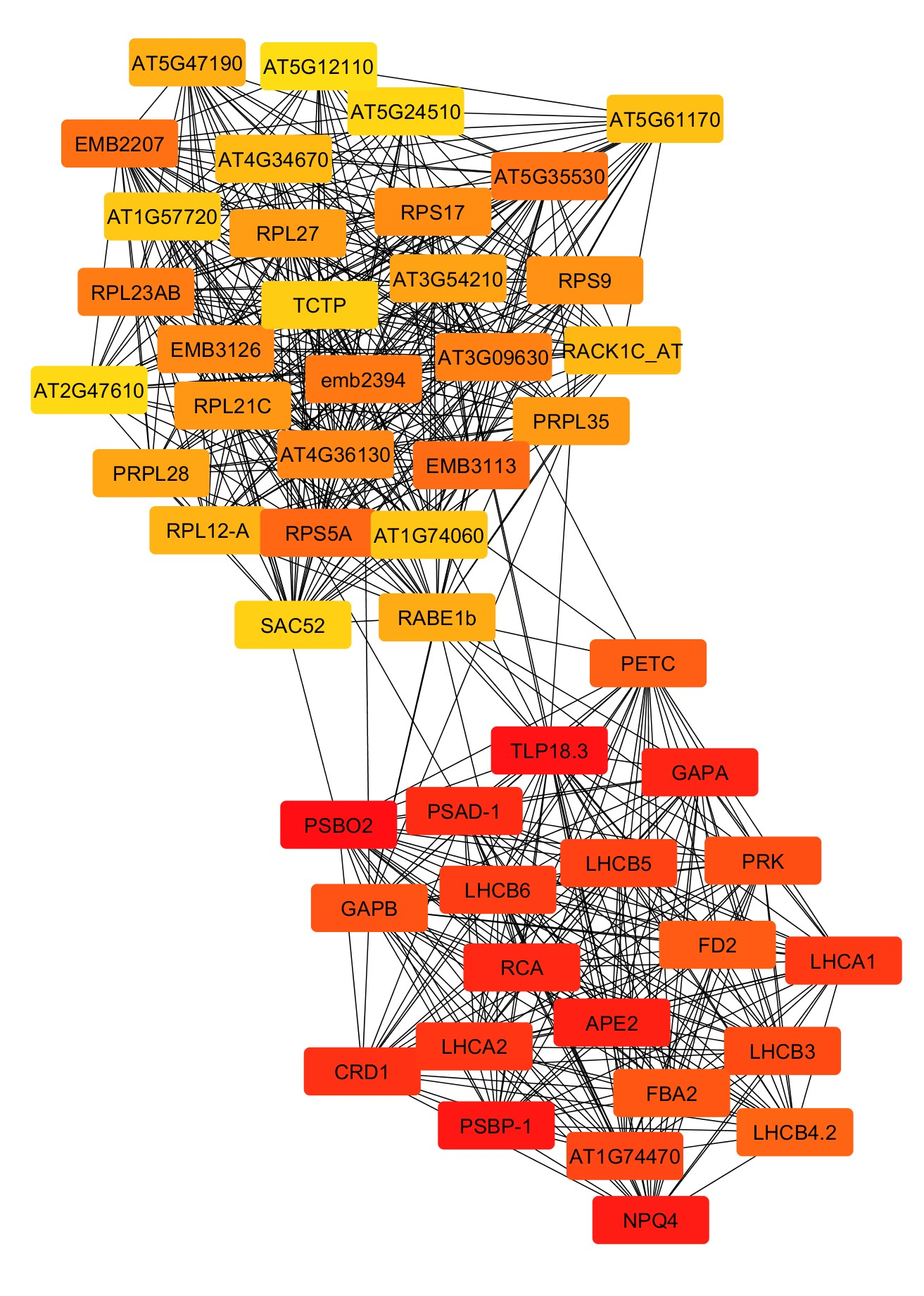

Supplement: S2 Fig — (TIF) [file pone.0287761.s002.tif]

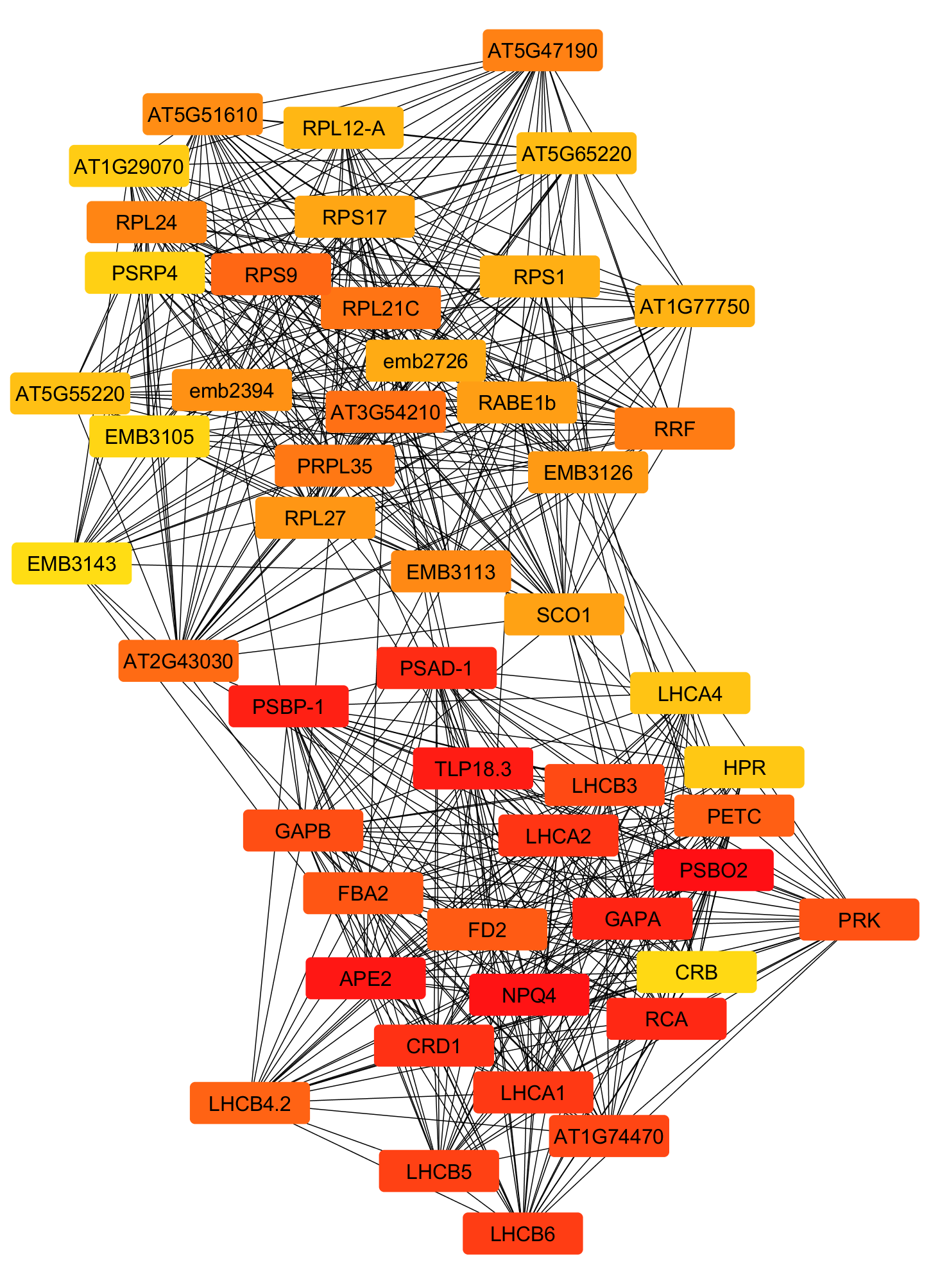

Supplement: S3 Fig — (TIF) [file pone.0287761.s003.tif]
