# Supplementary material for: A comprehensive analysis of transcriptomic data for comparison of plants with different photosynthetic pathways in response to drought stress
Source: PLoS One. 2023 Jun 27;18(6):e0287761. doi: 10.1371/journal.pone.0287761 (PMC10298789; doi:10.1371/journal.pone.0287761)
Supplement: Attachment — Submitted filename: Response to Editor and Reviewers.docx [file pone.0287761.s012.docx]

Dear Editor and Reviewers,

Thank you for your time and for providing valuable comments to improve our manuscript. Please find the answers to your questions and comments below. All parts that have been changed according to your comments are given in track changes in the revised manuscript. I hope that the revised file satisfies you.

**Response to Editor**

1. Please ensure that your manuscript meets PLOS ONE's style requirements

The revised manuscript was prepared based on PLOS ONE's style.

2. Please include a copy of Table 1 and 2 which you refer to in your text on page 8

We inserted Tables 1 and 2 in the revised manuscript.

Also a minor revision made in Figure 6 and Table S7-S8

**Response to Reviewer #1**

1. Extensive English editing before reviewing process.

We attempted to edit the entire text of the manuscript in order to make it more understandable.

**Response to Reviewer #2**

Reviewer: I suggest to address following two issues to further improve the quality of the manuscript.
1. The authors should describe the shortages/shortcomings of this research in “Discussion” section.

According to the suggestion of the respected reviewer, the limitations of our research were mentioned at the end of the discussion section.

2. The topics discussed in “Discussion” section need to be concentrated and focused on processes related to photosynthesis, especially on those impacting the behaviors of C3 and C4 plants treated by drought.

We tried not to limit our study to the main difference between C3 and C4 plants (C3 and C4 cycle) because this leads us to focus only on the effect of drought stress on the activity of C3 and C4 cycle enzymes. Our aim is to obtain comprehensive information about the overall response occurring in the leaves of these plants, which also includes photosynthesis. Because the response of C3 and C4 plants to stress conditions is not only related to photosynthesis but may be related to extensive metabolic pathways. Also, in the energy metabolism section, we tried to explain common and different changes in the expression of genes related to photosynthesis in both groups of plants (Response of light reactions and carbon fixation pathways). Therefore, it seems that it would be better to refer to the photosynthetic pathway in the title of the energy metabolism section, hence this change has been applied in the new version. Furthermore, to complete the photosynthesis section some information has been added.
