# Supplementary material for: A comprehensive analysis of transcriptomic data for comparison of plants with different photosynthetic pathways in response to drought stress
Source: PLoS One. 2023 Jun 27;18(6):e0287761. doi: 10.1371/journal.pone.0287761 (PMC10298789; doi:10.1371/journal.pone.0287761)
Supplement: Attachment — Submitted filename: response to veiwers (2).docx [file pone.0287761.s013.docx]

**Dear Editor and Reviewer**

Thank you for your time and for providing valuable comments to improve our manuscript. We have made modifications to our updated manuscript point by point according to your comments. Our response follows (the comments are in italics).

We hope that the revised file satisfies you.

**Response to Editor**

*Please review your reference list to ensure that it is complete and correct. If you have cited papers that have been retracted, please include the rationale for doing so in the manuscript text, or remove these references and replace them with relevant current references. Any changes to the reference list should be mentioned in the rebuttal letter that accompanies your revised manuscript. If you need to cite a retracted article, indicate the article’s retracted status in the References list and also include a citation and full reference for the retraction notice.*

**Response**: We checked all our references, the format of reference number 9 was modified and five references (31-35) were added in the previous revised manuscript (lines 431-437), one of which was forgotten (*), and this reference was also added in the new version. The number of references in the text and in the list of references has been updated, which can be traced in the Manuscript with Track Changes. On the other hand, for easier access to the references, the DOI of the articles was added.

9. Babraham Bioinformatics—FastQC A Quality Control tool for High Throughput Sequence Data [Internet]. [cited 2019 Feb 10]. Available from: <http://www.bioinformatics.babraham.ac.uk/projects/fastqc/>

31*. Lin YH, Huang LF, Hase T, Huang HE, Feng TY. Expression of plant ferredoxin-like protein (PFLP) enhances tolerance to heat stress in *Arabidopsis thaliana*. *New biotechnology*. 2015; 32(2): 235-242.‏ <https://doi.org/10.1016/j.nbt.2014.12.001>

32. Lehtimäki N, Lintala M, Allahverdiyeva Y, Aro EM, Mulo P. Drought stress-induced upregulation of components involved in ferredoxin-dependent cyclic electron transfer. J plant physiol. 2010;167(12): 1018-1022.‏ <https://doi.org/10.1016/j.jplph.2010.02.006>

33. Azzouz-Olden F, Hunt AG, Dinkins R. Transcriptome analysis of drought-tolerant sorghum genotype SC56 in response to water stress reveals an oxidative stress defense strategy. Mol Biol Rep. 2020;47: 3291-3303. <https://doi.org/10.1007/s11033-020-05396-5>

34. Fracasso A, Trindade LM, Amaducci S. Drought stress tolerance strategies revealed by RNA-Seq in two sorghum genotypes with contrasting WUE. BMC Plant Biol. 2016;16(1): 1-18. <https://doi.org/10.1186/s12870-016-0800-x>

35. Yang M, Geng M, Shen P, Chen X, Li Y, Wen X. Effect of post-silking drought stress on the expression profiles of genes involved in carbon and nitrogen metabolism during leaf senescence in maize (Zea mays L.). Plant Physiol Biochem. 2019;135: 304-309. <https://doi.org/10.1016/j.plaphy.2018.12.025>

**Response to Reviewer 2**

We would like to thank the reviewer for careful and thorough reading of this manuscript and for the thoughtful comments and constructive suggestions, which help to improve the quality of this manuscript. In addition, we appreciate the positive feedback from the reviewer. The responses are as below:

*Reviewer #2: The authors have well addressed the concerns raised by the reviewers. A suite of minor errors are needed to be corrected before the publish in the journal.*

*Line 57: “CO2” needs to be corrected, in which “2” is lowercased.*

**Response:** We have fixed the error.

*Line 82 : “The Pots” needs to be corrected by “The pots”.*

**Response:** We have fixed the error.

*Line 90: “drought stress NCBI Sequence”: a comma needs to added ahead of “NCBI Sequence”.*

**Response:** The correction has been made.

*Line 101: “S1 Table” should be “Table S1”.*

**Response:** We thank the reviewer for pointing this out. According to PLOS ONE's Supporting Information Citations "Format Supporting Information Citations as “S1 Fig”, “S1 Table”, etc.", we have cited this way.

*Line 141: cis-regulatory elements: “cis” herein needs to be italic.*

**Response:** The correction has been made.

*Line 154: “S2 Table” should be “Table S2”. Same as follow.*

**Response:** We thank the reviewer for pointing this out. According to PLOS ONE's Supporting Information Citations "Format Supporting Information Citations as S1 Fig, S1 Table, etc.", we have cited this way.

*Line 163: “Fig 1” should be “Fig. 1”. Same as follows.\*

**Response:** We thank the reviewer for pointing this out. According to PLOS ONE's Figure Citations "Cite figures as Fig 1, Fig 2, etc.", we have cited this way.

*Line 174: “Table 1 and 2” should be “Tables 1 and 2”.*

**Response:** The correction has been made.

*Lines 215-221: the capital letters needs to be lowercased. Same as in caption of Fig. 2.*

**Response:** The correction has been made*.*

*Line 256: “Porphyrin” should be “porphyrin”.*

**Response:** The correction has been made*.*

*Line 377: “are participate” should be “arparticipate”.*

**Response:** The correction has been made*.*

*Line 389: “miR-158” should be “miR158”.*

**Response:** We have fixed the error.

*Line 403: “Mun et al. [27]findings showed” should be “Mun et al. [27 showed”.*

**Response:** This observation is correct. We have changed.

*Line 483: “can also can cause” should be “can also cause”.*

**Response:** We have fixed the error.

*Line 535: “as an important” should be “an important”.*

**Response:** The correction has been made.
